# Sonic irrigant activation for root canal disinfection: power modes matter!

**DOI:** 10.1186/s12903-020-01088-5

**Published:** 2020-04-10

**Authors:** Florin Eggmann, Yvonne Vokac, Sigrun Eick, Klaus W. Neuhaus

**Affiliations:** 1 Department of Periodontology, Endodontology and Cariology, University Center for Dental Medicine, UZB, University of Basel, Mattenstrasse 40, CH-4056 Basel, Switzerland; 2 Private practice, Zurich, Switzerland; 3 Laboratory of Oral Microbiology, Department of Periodontology, School of Dental Medicine, University of Bern, Bern, Switzerland; 4 Department of Preventive, Restorative and Pediatric Dentistry, University of Bern, Bern, Switzerland

**Keywords:** Endodontic disinfection, Oral bacteria, Sonic activation, Needle irrigation, Sodium hypochlorite

## Abstract

**Background:**

Sonic irrigant activation has gained widespread popularity among general dentists and endodontists alike in recent years. This in vitro study aimed to evaluate the impact of three power modes of a sonic activation device (EDDY) on its antimicrobial effectiveness in infected root canals.

**Methods:**

The root canals of straight, human roots (*n* = 120) were prepared to size 40/.06. In a short-term infection experiment, the root canals were inoculated with different microbial species for three days. The following irrigation protocols, using 4 ml of normal saline as irrigant, were performed: negative control, manual rinsing, sonic irrigant activation at power modes “low”, “medium” and “high”. In a second, long-term experiment, testing the same irrigation protocols, inoculation lasted 21 days and sodium hypochlorite was used as irrigant. Sequential infection control samples were assessed using culture assays. The statistical analysis included one-way analysis of variance of log_10_-scaled counts of colony-forming units (CFU) with post-hoc comparisons using Bonferroni corrections and Chi^2^ tests (α = 0.05).

**Results:**

In the short-term experiment, the sonic irrigation protocols decreased the number of CFUs by 1.88 log_10_ units compared with the negative control (*p* < 0.001). The power modes “medium” and “high” achieved the most effective reduction of the microbial load. In the long-term experiment, microbial regrowth occurred after 7 days unless the device was used at its highest power setting.

**Conclusions:**

The power modes of the sonic irrigation device have a significant impact on the effectiveness for endodontic disinfection. The sonic irrigation device should always be used at the highest power setting in order to maximize its antimicrobial effectiveness.

## Background

Apical periodontitis is a disease caused by endodontic biofilms [1]. To achieve a favorable treatment outcome, it is crucial to eliminate or significantly reduce the microbial load within the root canal system and to prevent any recontamination after the treatment [2, 3]. However, the root canal system has an intricate anatomy, and root canal instrumentation with rotary files leaves, on average, 60% of the root canal wall surface untouched [4]. The uninstrumented portions of the root canal system and anatomical complexities, such as accessory canals, apical ramifications, fins and isthmus areas, can harbor tissue debris as well as microorganisms and their deleterious by-products in spite of thorough chemomechanical disinfection [1]. Supplementary irrigation methods such as irrigant activation techniques are therefore needed to enhance the elimination of endodontic biofilms [5].

Ultrasonic irrigant activation is the most commonly used supplementary irrigation method [6]. However, file-to-wall contact of ultrasonic instruments, all but unavoidable under clinical conditions, may lead to the inadvertent removal of small amounts of dentin and significant oscillation dampening [7, 8]. Compared with ultrasonic activation, sonic irrigation devices operate at lower frequencies. EDDY (VDW GmbH, Munich, Germany), a flexible, non-cutting polyamide tip that is coupled to an air scaler handpiece, is used with sonic activation at a frequency of 5000 Hz to 6000 Hz [9]. Sonic activation with soft polymer tips promises to be an alternative irrigant activation technique without the risk of unintentional dentin removal, [10] and in vitro evidence suggests that the antimicrobial efficacy of sonic activation with EDDY is, at least, on par with ultrasonic irrigant activation in straight and curved root canals [11].

Previous studies on the disinfection effectiveness of irrigation acitivation with EDDY employed the sonic device at the highest power mode of the air scaler handpiece [11]. Some dental practitioners may, however, use EDDY with air scaler handpieces set at lower power modes in order to reduce irrigant spray mist during activation (VDW GmbH, oral communication). In ultrasonic irrigant activation, the power setting influences the file oscillation amplitude, which, in turn, has a pronounced effect on irrigant streaming and cleaning effectiveness [12]. Whether or not different power settings have a similar impact on the effectiveness of sonic irrigant activation has not yet been investigated.

The aim of this in vitro study was therefore to assess, through microbiological testing, if different power modes affect the antimicrobial effectiveness of sonic irrigation activation with the EDDY device.

## Methods

### Selection and preparation of roots

One hundred and twenty mature, straight roots (root curvature radius < 15° [13]) of maxillary front teeth, single-rooted maxillary premolars, and palatal roots of maxillary molars were selected from a pool of extracted human permanent teeth stored in a 1% aqueous solution of chloramine-T. The roots had neither resorptive defects nor fracture lines. The local Ethics Committee denied the need for a formal approval for using irreversibly anonymized teeth and issued a written declaration of no-objection. All donors had given their verbal informed consent to the use of their extracted, anonymized tooth/teeth for research purposes.

To remove the periodontal tissue, the roots were immersed in 3% (w/v %) sodium hypochlorite (NaOCl) for 10 min. The working lengths were determined using a 10/.02 K file (C-PILOT, VDW GmbH, Munich, Germany). The working length was defined as 0.5 mm shorter than the length at which the instrument was first visible at the apical foramen under 2.5x magnification. After confirmation of canal width by reassuring passive fit and gauging with a 20./02 K file, root canal shaping was performed with a reciprocating single-file system (RECIPROC #40, VDW GmbH); the rinsing solutions, used alternately during the shaping of the root canals, were 3% (w/v %) NaOCl and 17% (w/v %) ethylenediaminetetraacetic acid.

After shaping the root canals, the external root surfaces of the apices were conditioned for 15 s with a phosphoric acid etchant (Ultra-Etch, Ultradent Products Inc. South Jordan, UT, USA), which was rinsed off with water for 15 s. A multimode adhesive (Scotchbond Universal, 3 M Oral Care, St. Paul, MN, USA) was applied actively with a microbrush for 20 s, then the adhesive was gently air dried for 5 s. The apices were closed with resin-based composite (Telio CS Inlay, Ivoclar Vivadent AG, Schaan, Principality of Liechtenstein). Light curing of the adhesive and the resin-based composite was performed with a polymerization light at an irradiance of 1200 mW/cm^2^ (Bluephase [high power mode], Ivoclar Vivadent AG) and as short a distance between the tip of the light probe and the adhesive/resin-based composite as feasible for 10 s and 30 s, respectively. The roots were stored in deionized water at 4 °C until further use. Prior to the microbial contamination procedure, the roots were autoclaved in water for 20 min at 121 °C (Laboklav ECO, SHB Steriltechnik AG, Detzel Schloss/Satuelle, Germany).

### Short-term infection experiment

#### Microbial contamination

The following microorganisms were separately precultured and suspended for 18 h:

1. *Enterococcus faecalis* ATCC 29212
2. *Candida albicans* ATCC 76615
3. *Streptococcus gordonii* ATCC 10558 and *Actinomyces oris* ATCC 43146

Thirty autoclaved roots were randomly allocated to each group of microorganisms and inoculated for three days at 37 °C. Groups 1 and 2 were cultured under aerobic conditions. Group 3 was cultured with 10% CO_2_. Tryptic soy agar (TSA) plates with 5% sheep blood were used for the cultivation of the microorganisms. Then, suspensions of *E. faecalis* ATCC 29212 and *C. albicans* ATCC 76615 were prepared in a 0.9% sodium chloride solution (McFarland 4), which was then diluted, at a ratio of 1:9, with a brain-heart infusion broth (Oxoid Limited, Basingstoke, UK). Wilkins-Chalgren anaerobe broth (Oxoid Limited) supplemented with 5 mg/l β-nicotinamide adenine dinucleotide sodium salt (NAD) (Sigma-Aldrich, St. Louis, MO, USA) was used for the suspension of *S. gordonii* ATCC 10558 and *A. oris* ATCC 43146 (mixed at a ratio of 1:2). The nutrient broths were renewed daily. At the beginning of the microbiological experiments, sterility was monitored by sampling and culturing on TSA plates under both aerobic and anaerobic conditions as described above. Cultivation was performed over 4 days.

#### Irrigation protocols

Normal saline, i.e., a solution of 0.9% w/v of sodium chloride, was used as irrigation solution. The root canals, inoculated with microorganisms, were subjected to the following irrigation protocols:

1. Negative control (*n* = 6): no treatment
2. Manual rinsing (n = 6): manual irrigation, 4 ml irrigation solution
3. Power mode “low” (*n* = 6): sonic irrigant activation with a polyamide tip (6000 Hz; EDDY, VDW GmbH) coupled to an air scaler (SONICflex, KaVo Dental GmbH, Biberach, Germany), 3 × 20 s, 4 ml irrigation solution (3 ml for continuous irrigation during activation, 1 ml as a final rinse)
4. Power mode “medium” (*n* = 6): sonic irrigant activation with a polyamide tip (6000 Hz; EDDY, VDW GmbH) coupled to an air scaler (SONICflex, KaVo Dental GmbH), 3 × 20 s, 4 ml irrigation solution (3 ml for continuous irrigation during activation, 1 ml as a final rinse)
5. Power mode high (n = 6): sonic irrigant activation with a polyamide tip (6000 Hz; EDDY, VDW GmbH) coupled to an air scaler (SONICflex, KaVo Dental GmbH), 3 × 20 s, 4 ml irrigation solution (3 ml for continuous irrigation during activation, 1 ml as a final rinse)

The power modes “low”, “medium” and “high” corresponded to the markings “1”, “2” and “3”, respectively, on the SONICflex air scaler handpiece. The respective amplitudes within the excitation unit of the handpiece were 120 ± 15 μm, 160 ± 15 μm and 240 ± 15 μm according the manufacturer (Ulrike Nagorr, KaVo Dental GmbH, written communication). All irrigants were delivered by single-use syringes with a capacity of 10 ml (Omnifix Luer Solo, B. Braun Medical AG, Sempach, Switzerland). In the manual rinsing group (group 2) and for the final rinse in groups 3–5, side-vented Gauge 30 irrigation needles (Irrigation Probe, KerrHawe SA, Bioggio, Switzerland) were inserted to working length. The sonic irrigation tips, likewise, were placed at working length. The sterile single-use EDDY tips were replaced for the irrigant activation of each root. For continuous irrigation during sonic activation, the side-vented needle was placed within the coronal third of the root canal. All endodontic procedures were performed by one operator (YV) throughout the study, and all irrigants that were used were at room temperature.

#### Counting of colony-forming units

Sterile paper points (R40, RECIPROC Paper Points, VDW GmbH) that matched the taper of the file used for root canal preparation were placed in each root at working length for 30 s. The paper points were then placed in tubes filled with 1 ml 0.9% NaCl. The tubes were vortexed for 20 s and sonicated in an ultrasonic bath before 0.1 ml of the solution was dispersed on TSA plates. Incubation of *E. faecalis* ATCC 29212 and *C. albicans* ATCC 76615 was performed for 2 days. Incubation of *S. gordonii* ATCC 10558 and *A. oris* ATCC 43146 lasted 7 days. After the incubation periods, the number of colony-forming units was determined by using an automated colony counter (aCOLyte, Synbiosis, Cambridge, UK).

### Long-term infection experiment

Thirty autoclaved roots were inoculated with *S. gordonii* ATCC 10558 and *A. oris* ATCC 43146. The incubation period lasted three weeks at 37 °C with 10% of CO_2_. Apart from the irrigation solution, the irrigation protocols were analogous to those in the short-term infection experiment. NaOCl (1.5% w/v) was used as irrigation solution instead of 0.9% NaCl. One ml of 0.9% NaCl was only used for the final rise. A rest period of 60 s was added after NaOCl delivery in the manual rinsing group to standardize irrigant contact time across all groups. Immediately after the irrigation protocol, a microbiological sample was collected with a sterile paper point as described above. Thereafter, the root canals were filled with nutrient broth and they were incubated at 37 °C as described above. Microbiological samples were collected from the root canals after 3, 5 and 7 days. The samples were processed following the same procedures as in the short-term infection experiment. A qualitative dichotomous analysis was performed to assess the presence of viable microorganisms after incubation periods of 0, 1, 4 and 7 days.

### Statistical analysis

Statistical analyses were performed with SPSS 24.0 (IBM SPSS Statistics, Chicago, IL, USA). Statistical analyses based on log_10_-scaled microbial counts (total colony forming units ([CFU]) for the short-term experiment. Data were compared using a one-way analysis of variance (ANOVA) with post-hoc comparisons of groups using Bonferroni corrections. For the long-term infection experiment, the Chi^2^ test was employed. The level of significance was set at α = 0.05.

## Results

### Short-term infection experiment

No contamination of the samples occurred throughout the duration of the experiment. All irrigation protocols with sonic activation significantly reduced the number of viable microorganisms, *E. faecalis* and *Candida albicans*. The order of magnitude of the reduction of CFUs of *E. faecalis* was 1.82 log_10_ units for all sonic activation protocols (groups 3–5) compared with the negative control (group 1) (*p* < 0.001), and 1.20 log_10_ units compared with the manual irrigation group (group 2) (*p* ≤ 0.006). No statistically significant difference was observed between the manual irrigation protocol and the negative control group (*p* = 0.37). The different power modes showed no statistically significant differences (*p* = 1.00). The order of magnitude of the reduction of CFUs of *Candida albicans* was 1.83 log_10_ units for all sonic activation protocols (groups 3–5) compared with the negative control (group 1) (*p* < 0.001), and 1.31 log_10_ units compared with the manual irrigation group (group 2) (p < 0.001). Statistically significant differences were neither observed between the manual irrigation protocol and the negative control group (*p* = 0.93) nor between the different power modes (*p* = 1.00).

**Fig. 1:**
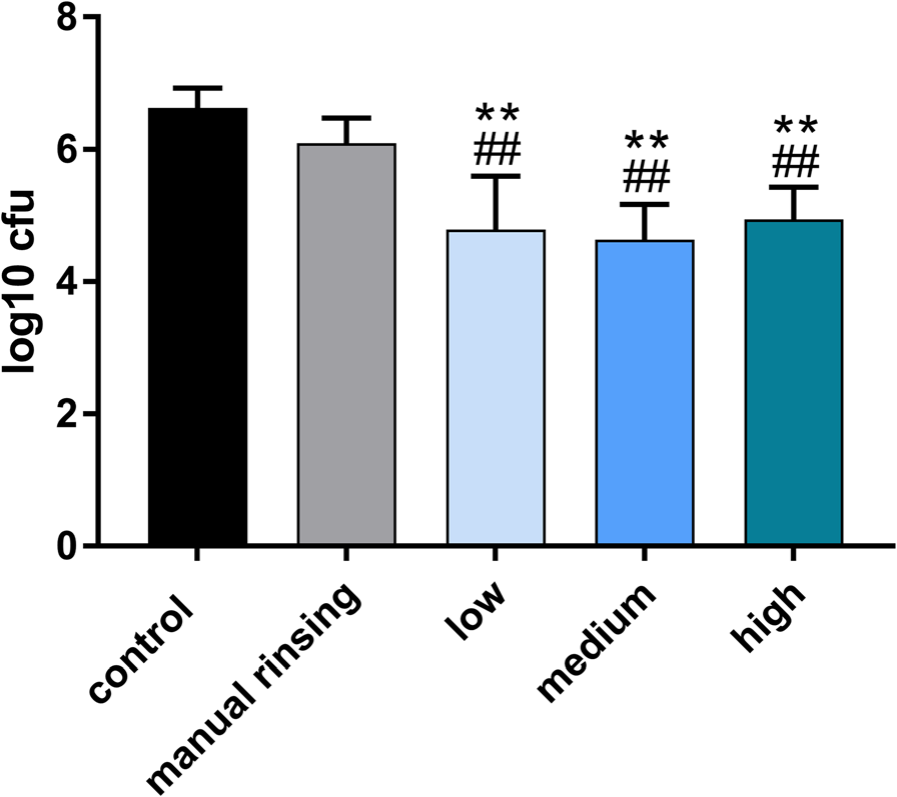
Log_10_ units of CFUs of *Candida albicans* after different irrigation protocols. “Low”, “medium” and “high” refer to respective the power mode of the air scaler handpiece used for sonic irrigation with the EDDY device. **,## indicate *p* < 0.05

**Fig. 2:**
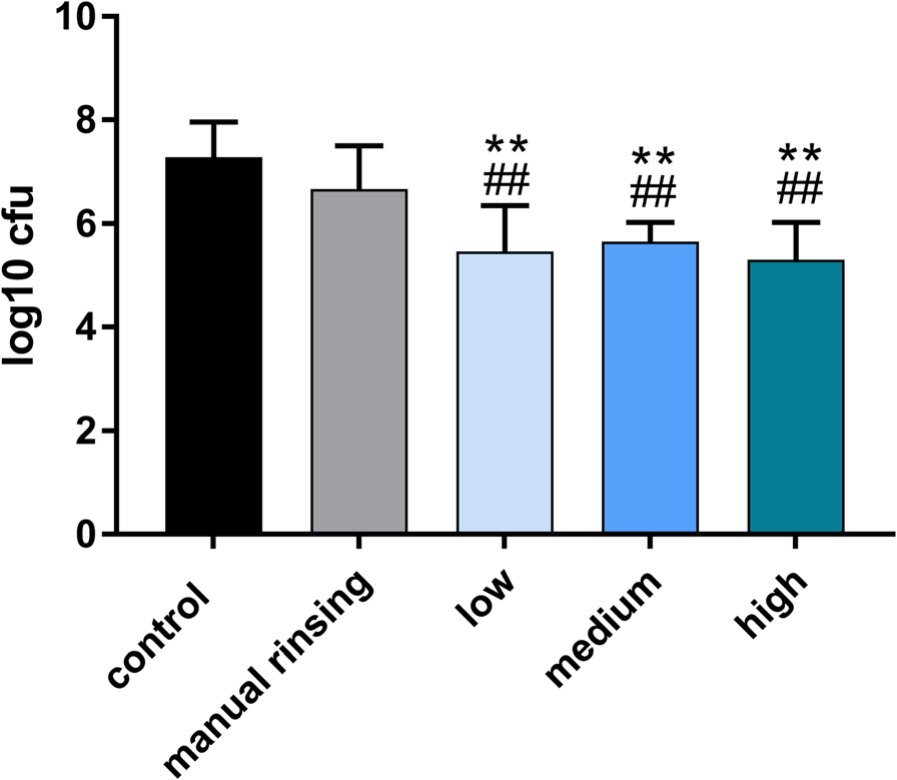
Log_10_ units of CFUs of *E. faecalis* after different irrigation protocols. “Low”, “medium” and “high” refer to respective the power mode of the air scaler handpiece used for sonic irrigation with the EDDY device. **,## indicate p < 0.05

**Fig. 3:**
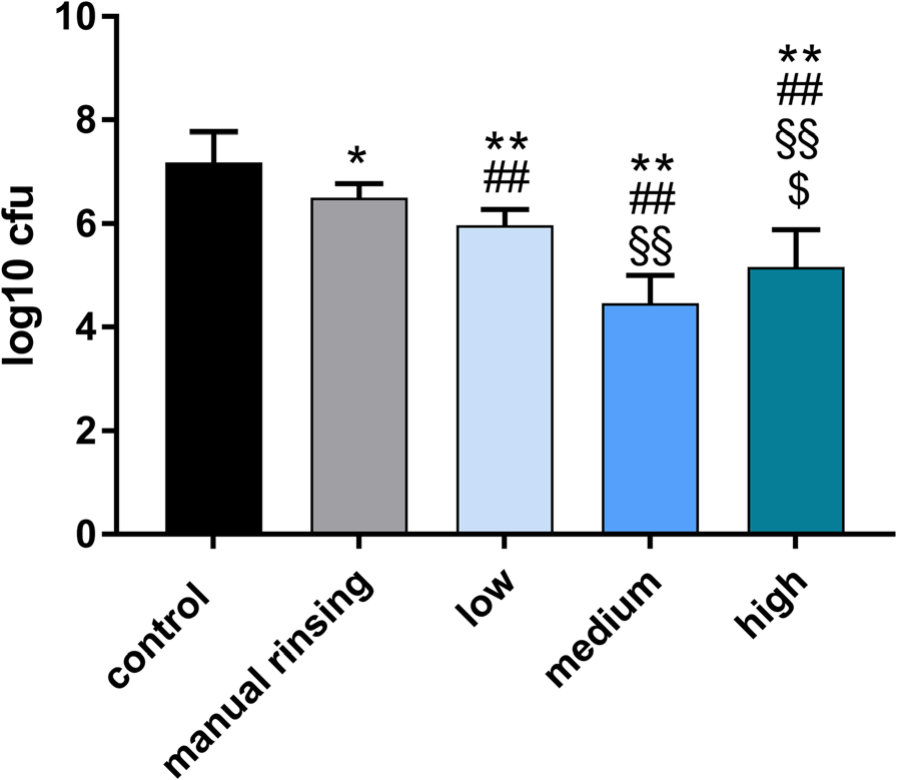
Log_10_ units of CFUs of *S. gordonii* and *A. oris* after different irrigation protocols. “Low”, “medium” and “high” refer to respective the power mode of the air scaler handpiece used for sonic irrigation with the EDDY device. **,##,§§,$ indicate *p* < 0.05

In comparison with the negative control group, all irrigation protocols significantly reduced the number of *S. gordonii* and *A. oris*: the order of magnitude of the reduction of CFUs was 2.72 log_10_ units for the sonic activation power mode “medium” (*p* < 0.001), 2.03 log_10_ units for the sonic activation power mode “high” (p < 0.001) and 1.21 log_10_ units for the sonic activation power mode “low” (p < 0.001). The manual irrigation protocol reduced the number of CFUs by 0.69 log_10_ units compared with the negative control group (*p* = 0.022). Overall, the sonic irrigation protocols decreased the number of CFUs by 1.30 log_10_ units compared with the manual irrigation protocol. Among the sonic irrigation protocols, the power mode “medium” obtained the most substantial CFU reduction: it decreased the number of CFUs by 1.51 log_10_ units compared with the power mode “low” (*p* < 0.001) and by 0.69 log_10_ units compared with the power mode “high” (*p* = 0.020). The power mode “high” reduced the number of CFUs by 0.82 log_10_ units compared with the power mode “low” (*p* < 0.004), Figs. 1, 2 and 3 show detailed results of the short-term infection experiment.

### Long-term infection experiment

**Fig. 4:**
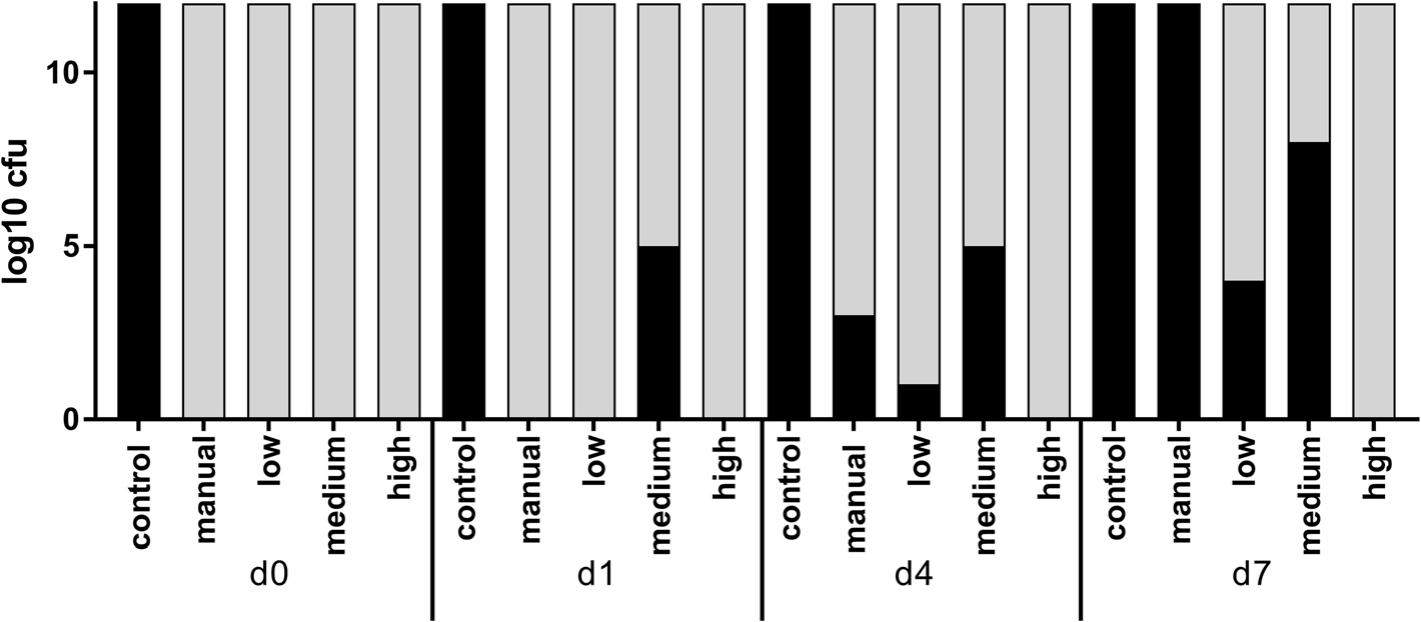
Bacterial regrowth (log_10_ units of CFUs, black bars) after irrigation with 1.5% NaOCl (d0, day 0) and after re-incubation and provision of nutrient broth (d1, day 1; d4, day 4, d7, day 7). “Low”, “medium” and “high” refer to respective the power mode of the air scaler handpiece used for sonic irrigation with the EDDY device. “Manual” stands for the manual rinsing group

No contamination of the samples occurred throughout the duration of the experiment. After three weeks of incubation with *S. gordonii* and *A. oris,* no bacteria could be cultivated from the samples taken immediately after irrigation with NaOCl. The addition of nutrient broth and incubation at 37 °C led to bacterial regrowth in all root canals after 7 days except for those that had been irrigated with sonic activation at the highest intensity (power mode “high”). Detailed results of the long-term infection experiment are provided in Fig. 4.

## Discussion

This in vitro investigation showed that the power modes of a sonic irrigation device have a significant impact on the antimicrobial efficacy in root canals. In the short-term infection experiment, the two higher power modes achieved a more substantial reduction of viable microorganisms than the lowest power mode. In the long-term infection experiment, bacterial regrowth was completely stunted exclusively in root canals that had been irrigated with the sonic irrigation device employed at the highest power setting.

Recent in vitro studies have shown that, compared with manual irrigation, the sonic irrigation device EDDY improves the dentin debris removal from artificial canal irregularities [14], the removal of calcium hydroxide from artificial grooves in the root canal of maxillary incisors [9], the removal of remnants of sealer and guttapercha [15] and the median dye penetration depths in dentinal tubules of apical root sections [16]. However, considering the pivotal etiopathogenetic role of endodontic biofilms in periapical disease, the reduction of the microbial load within root canal systems is arguably the most relevant surrogate marker in experimental irrigation studies [17]. Although microbiological studies are fraught with some methodological difficulties, the endodontic microbiological status can, at least to some degree, predict healing of apical periodontitis [18].

The setup of the present study is comparable to a previous in vitro study which indicated that, in straight and curved root canals, sonic irrigation with EDDY surpasses manual syringe irrigation in terms of antibacterial efficacy [11]. However, an in-depth comparison of the results of that study and the present investigation reveals that the order of magnitude of CFU reductions are, in some cases, more than one logarithmic step apart even though the overall trend is qualitatively similar in both studies. For instance, Neuhaus et al. [11] reported median log_10_ CFU counts of 4.03 for the *S. gordonii* and *A. oris* co-culture, 2.98 for *E. faecalis* and 1.7 for *C. albicans* after sonically activated irrigation of straight root canals. In the present investigation, on the other hand, the log_10_ CFU counts in the group with sonic irrigant activation at the highest power setting were 5.16 for the *S. gordonii* and *A. oris* co-culture, 5.30 for *E. faecalis* and 4.94 for *C. albicans* in the short-term infection experiment.

In this study, microorganisms were used that are frequently found in infected root canal and that have the potential to invade radicular dentin [19]. The in vitro study of Neuhaus et al. [11] found regrowth of *C. albicans* and *E. faecalis* in all samples after seven days, i.e. some viable microorganisms that evaded detection – presumably in dentinal tubules - immediately after irrigation remained in the root canal system. No microbial regrowth occurred in the long-term infection experiment in the roots that were infected with *S. gordonii* and *A. oris* [11]. In that study, however, the sonic irrigation was always performed at the highest power setting [11]. The present investigation revealed that microbial regrowth of *S. gordonii* and *A. oris* occurred in all samples apart from those that had been sonically irrigated at the highest power setting. The irrigation protocols and microbiological assays were carried out in an analogous manner in both studies. However, in the study of Neuhaus et al. [11], the root canals were shaped with a reciprocating file size 25/.08 and both straight and curved roots were included in the long-term infection experiment. Contrary to this, the tooth sample of the present study included only straight roots and the root canals were prepared with a reciprocating file size 40/.06. The increased apical enlargement and the absence of curved roots provided favorable conditions for improved irrigant delivery to the apical part of the main root canal and more unimpeded oscillation of the EDDY tip in the present study [7, 20, 21]. These advantages notwithstanding, the EDDY device employed at the two lower power settings was insufficiently efficacious in eliminating residual microorganisms. This underlines that sonic irrigant activation should be performed at the highest power mode even if the conditions for root canal disinfection seem favorable.

Data from a previous in vitro study which assessed how effective sonic irrigant activation with EDDY was against *E. faecalis* suggest that EDDY is more efficient than syringe needle irrigation against intratubular bacterial in all portions of the root [22]. Yet, EDDY does not maintain its antibacterial superiority in dentinal tubules beyond a depth of 100 μm [22]. Considering the limited reach of sonically activated irrigants in dentinal tubules, it has been argued that the concentration of NaOCl is more important than sonic irrigant activation to eliminate bacteria residing in dentinal tubules [22]. The present investigation confirms that sonic irrigant activation with EDDY, employed at the highest power setting, outperforms the antimicrobial effect of manual syringe irrigation but, in contrast to the studies of Hage et al. [23] and Zeng et al. [22], no viable microorganism were found even after an extended re-incubation period of seven days. In addition to different microbiological assays and variations in the irrigation protocols, the different makeup of tooth samples may account, at least to some degree, for these conflicting microbiological results. In the studies of Hage et al. [23] and Zeng et al. [22] exclusively single-rooted premolars were used whereas the sample of the present study included anterior teeth, premolars and palatal roots of maxillary molars. Premolars are frequently extracted in adolescence and early adulthood for orthodontic reasons. The anterior teeth and molars contained in the sample of the present study, by contrast, were likely to come predominately from older patients with tooth loos resulting from periodontal disease. It is thus reasonable to assume that the average age of the teeth in the samples of these studies were different. The degree of root dentin sclerosis increases with advancing age, and consequently bacterial infection of dentinal tubules is less pronounced in older teeth [17, 24]. This may influence the antimicrobial effect of irrigation protocols [17, 24]. A tentative conclusion that can be drawn based on the results of the studies which assessed the antibacterial efficacy of the EDDY device is that sonic irrigant activation is advantageous compared with manual syringe irrigation but cases with severe intratubular infection may still require adjunct measures such as the use of higher NaOCl concentrations and prolonged exposure times [22, 23, 25].

In the present investigation, an established in vitro model with extracted teeth with closed-end root canals was used in order to have a standardized and reproducible setup and to replicate in vivo scenarios as closely as possible [5, 11]. It is important to give due consideration to the inherent strengths and limitations of this model and the microbiological methods that were employed.

Decoronated roots were used for this in vitro study in order to increase the level of standardization and to ensure a straightline access and adequate visual control of the irrigation needles and EDDY tips during the irrigation procedures. However, the use of decoronated roots might have influenced the irrigation protocols and thus the microbiological findings. For example, coronal tooth substance or restoration material can sometimes compromise the straight access to the root canal orifice and the resulting interference between the crown and the EDDY tip is likely to affect the oscillation behavior of the latter.

The root curvature affects the effectiveness of activation methods that rely on the insertion of an instrument in the root canal [5, 20]. To decrease the potential for confounding, the study material comprised exclusively of roots with no pronounced curvatures. Moreover, as in the majority of in vitro studies, single roots with a simple anatomy were used in the present investigation. Micro-computed tomography data suggest, however, that in the complex mesial root canal systems of mandibular molars sonic irrigant activation does not improve the removal of accumulated hard-tissue debris compared with conventional manual irrigation [10]. Whether or not the same holds true for the antimicrobial efficacy remains currently unclear. Interestingly, a recent in vitro study that used a standardized isthmus model reported that laser-activated irrigation and sonic irrigant activation with EDDY achieved the most thorough removal of a biofilm-mimicking hydrogel [26]. Corroborating microbiological evidence for this finding is, however, lacking for the time being. Further studies are, therefore, necessary to evaluate if the results of the present investigation are replicable in teeth with multiple root canals, curved roots or more complex root canal anatomies.

Endodontic infections are caused by multispecies biofilms. This in vitro investigation, however, used microbial monocultures and two-species co-cultures, consisting of microorganisms commonly found in infected root canals, to assess the effectiveness of different irrigation protocols. Therefore, the results of this study are not directly translatable to situations where multispecies biofilm are present. Biofilm formation and maturity have a determining influence on the antimicrobial effectiveness of irrigation protocols [5]. While biofilms grown from oral bacteria are quite susceptible to antimicrobial agents within the first two weeks of growth, more mature biofilms are more resistant and so the same antimicrobial agents are considerably less effective after three weeks [27]. The present study used two different maturation timelines for the biofilm models in order to take account of the different degrees in biofilm resistance. In line with methodological recommendations, the microbial purity of the contaminated roots was strictly monitored throughout the experiments to rule out any contamination [5]. The extent of microbial growth on the root canal walls and in dentinal tubules was not assessed by microscopic imaging or histology. Nevertheless, the microbiological results of negative controls provided a framework that allowed to quantitatively evaluate the effect of different irrigation protocols.

Conclusive evidence indicating which microbiological sampling method is best suited for irrigation studies is currently lacking [5]. Paper points allow straightforward sampling from within the main root canal lumen whereas sampling methods using dentin debris may yield more illuminating insight into dentinal tubule disinfection [5]. The microbiological approach of the present investigation was developed to remedy some of the shortcomings of paper point sampling [28]. As a response to a scarcity of nutrients and other adverse conditions for life, some microorganisms enter a viable but non-culturable state. The reintroduction of nutrients recovers the metabolic activity, albeit quite slowly for some oral bacteria, which can take up to three days for their reactivation [29]. The provision of nutrient broth and sequential microbiological sampling over an incubation period that lasted one week ensured that slow and lagged microbial regrowth was detectable in the present investigation.

Syringe irrigation was chosen as control group because it is the most popular irrigation method among general dental practitioners and endodontists alike [6, 30]. To overcome some of the limitations of syringe irrigation with side-vented needles [21, 31], the root canals in this study were shaped with a file that achieved an apical enlargement of an ISO size 40 and a taper of .06 along the most apical millimeters. In addition, small needles, Gauge size 30, that allowed placement at working length without binding were used to facilitate adequate irrigant delivery to the full extent of the main root canal and to improve irrigant exchange through an effective reverse flow [9, 31–33]. It is, however, important to bear in mind that the irrigant flow of side-vented needles is nonuniform and the mechanical cleaning effect thus tends to concentrate on the areas of the root canal walls next to the vent [33]. The orientation of the side-vent during irrigation was not monitored in the present investigation. Furthermore, the tip of the needle remained at working length for the whole duration of the irrigation even though up and down movement of needles is likely to be advantageous in terms of mechanical cleaning. However, as standardized vertical movements of the needles were not possible in the setup of this study, irrigation with the needle static at working length was deemed the preferable approach for better comparability. The sonic irrigation tip, too, was inserted to working length and remained there during activation despite the fact that the manufacturer recommends moving the sonic irrigation tip carefully up and down with vertical motions. The manufacturer’s recommendation was deliberately disregarded because, again, manually carrying out vertical motions in a standardized way was considered unfeasible.

The irrigant that was used in the short-term infection experiment differed from the one used in the long-term infection experiment. This restricts the comparability of the results of the two experiments and needs to be taken into consideration when contrasting these results. In the short-term infection experiment, normal saline was used as irrigant in order to asess the microbial load reduction that can be achieved through fluid dynamics alone. Biofilms at this stage of maturation are highly susceptible to potent antimicrobial irrigants such as sodium hypochlorite and, consequently, relevant differences in the effectiveness of different irrigations protocols would likely not have been detectable with culture assays if sodium hypochlorite had been used. It is essential to bear in mind, though, that normal saline was chosen simply to avoid falling below the microbiological detection threshold. In clinical cases, it is crucial to use an effective antimicrobial and proteolytic agent as main irrigant regardless of the stage of root canal infection.

Clinical treatment trials are needed to assess whether the differences in antimicrobial efficacy observed in the present investigation have a measurable impact on patient-centered outcomes such as healing of apical periodontitis. The predictive validity of in vitro irrigation studies for clinical outcome parameters is limited. For instance, although a substantial body of in vitro evidence suggests that ultrasonic irrigant activation is more effective than syringe irrigation, there is a paucity of clinical evidence for the benefit of ultrasonic irrigant activation in primary root canal treatment of teeth with a single root canal [17].

## Conclusions

Within the limitations of this in vitro study, it can be concluded that the power modes of the sonic irrigation device EDDY have a significant impact on its effectiveness for root canal disinfection. Lower power settings compromise the antimicrobial effectiveness and they need, therefore, to be avoided. In order to reduce the microbial load within the root canal system as effectively as feasible, the handpiece that is used to drive the sonic irrigation tip ought to be employed at its highest power setting at all times.

## Data Availability

The datasets used and/or analyzed during the current study are available from the corresponding author on reasonable request.

## Abbreviations

ANOVA: Analysis of variance
*A. oris*: *Actinomyces oris*
*C. albicans*: *Candida albicans*
CFU: Total colony forming units
*E. faecalis*: *Enterococcus faecalis*
NAD: β-nicotinamide adenine dinucleotide sodium salt
NaOCl: Sodium hypochlorite
*S. gordonii*: *Streptococcus gordonii*
TSA: Tryptic soy agar
